# Chemical antipredator defence is linked to higher extinction risk

**DOI:** 10.1098/rsos.160681

**Published:** 2016-11-23

**Authors:** Kevin Arbuckle

**Affiliations:** 1 Department of Biosciences, College of Science, Swansea University, Swansea SA2 8PP, UK; 2 Department of Evolution, Ecology and Behaviour, Biosciences Building, University of Liverpool, Crown Street, Liverpool, Merseyside L69 7ZB, UK

**Keywords:** conservation status, antipredator mechanisms, evolutionary ecology, biodiversity, phylogenetic comparative methods

## Abstract

Many attributes of species may be linked to contemporary extinction risk, though some such traits remain untested despite suggestions that they may be important. Here, I test whether a trait associated with higher background extinction rates, chemical antipredator defence, is also associated with current extinction risk, using amphibians as a model system—a group facing global population declines. I find that chemically defended species are approximately 60% more likely to be threatened than species without chemical defence, although the severity of the contemporary extinction risk may not relate to chemical defence. The results confirm that background and contemporary extinction rates can be predicted from the same traits, at least in certain cases. This suggests that associations between extinction risk and phenotypic traits can be temporally stable over long periods. The results also provide novel insights into the relevance of antipredator defences for species subject to conservation concerns.

## 1. Introduction

In relation to efforts in biological conservation, there is a growing expectation that decisions about allocation of limited resources (such as finances and personnel) have a firm evidence base underlying them [1,2]. Typically, such decisions are aimed to benefit those species deemed to be at higher risk of extinction, as often determined by the International Union for Conservation of Nature's (IUCN) Red List categories [3]. However, the IUCN currently lists 16.5% of species (12 609 of 76 199 species) as data deficient [4], and many species are not yet included on the list at all. Moreover, attempts to predict the conservation status of data-deficient species have suggested that a high proportion of these are likely to be threatened [5,6].

As a result of this lack of information on the conservation status of many species, several studies have attempted to investigate potential correlates of extinction risk (mostly using Red List status as a proxy) from phenotypic traits [6–14]. Certain traits are either known or suspected to impact on factors such as mortality rates [15,16] or the ability of populations to recover after declines [17,18], and therefore are potential predictors of extinction risk. It is therefore important that we know which traits predict conservation status, and how well they do so, as we can use these to inform resource allocation decisions for conservation.

Analogous to attempts to predict contemporary extinction risk, there has been a recent surge of interest in identifying traits that correlate with macroevolutionary (‘background’) diversification rates. Such studies have found that a wide range of traits influence the net diversification, speciation and extinction rates of many groups of organisms [19–23]. Traits that are linked to increased background extinction rates may, via similar mechanisms, also lead to a greater contemporary extinction risk; however, studies looking across temporal scales are lacking. It is perhaps unsurprising that this area has been neglected, because different threats are likely to be in operation now than throughout evolutionary history. For instance, many threats currently facing biodiversity today are anthropogenic in nature [4,24,25], such as rapid habitat destruction, exploitation or pollutants. As such, different traits may be important in mediating the extinction risk of species today than would have been important in the past. Nevertheless, the effect of a trait on extinction risk may operate by general mechanisms that make it possible to carry over conclusions about background extinction rates to contemporary extinction risk. For example, species with a slow life history may find it difficult to recover populations after declines when compared with similar species with faster life histories [10,12], regardless of the cause of the decline. At the very least, studies of trait-dependent diversification can lead to testable hypotheses that may point to traits that predict present-day extinction risk.

Amphibians are currently considered the most threatened vertebrate taxon and are experiencing population declines globally for both anthropogenic and enigmatic reasons [4,26,27]. Consequently, a range of traits have been evaluated as predictors of extinction risk in this group [10,11,28], and also as drivers of evolutionary diversification patterns [19,21,29,30]. A recent macroevolutionary study revealed that chemical antipredator defence is associated with higher extinction [30], a trait not previously considered in studies of contemporary extinction risk.

I have previously suggested three main possibilities to explain higher background extinction rates in chemically defended amphibians [30]: (i) chemical defence is energetically costly, (ii) chemical defence allows shifts to ‘marginal’ (low carrying capacity) habitats, which are intrinsically more vulnerable and (iii) chemical defence is associated with slow life histories, which damages the recovery potential of populations after declines. To these could be added the possibility that well-defended species face particularly strong predation pressure and that this could then lead to higher extinction rates. However, although high risk of predation is likely to explain the initial evolution of highly effective defences owing to coevolutionary arms races [31], once evolved such defences are typically associated with lower predation rates [32]. Although a serious attempt to distinguish between these hypotheses is beyond the scope of the current article, assessing whether similar patterns are found over widely disparate timescales may provide some insights. If chemical defence is found to be associated with both background and contemporary extinction risks, then it suggests that more general underlying mechanisms that make population recovery difficult after declines (whatever the cause of the decline) are more plausible. Understanding trait-associated patterns of extinction risk across different timescales may therefore lead to a more nuanced perspective on what type of historically adaptive traits we might expect to remain adaptive in the face of current anthropogenic threats.

In this paper, I test the prediction that chemically defended amphibian species also face a greater current risk of extinction, and in doing so test whether trait-dependent patterns of extinction risk are temporally stable and whether this is relevant to setting conservation priorities.

## 2. Methods

### 2.1. Data collection

Data on the presence or absence of chemical defence in 857 amphibian species were extracted from reference [30]. Briefly, this dataset was assembled from literature searches, using a conservative approach in which data were only recorded for each species if it had been investigated and found to either possess or lack a chemical defence. If information was not available for that given species no data were recorded and, consequently, species included in this study are known to either possess or lack a chemical antipredator defence. Further details on the collation of this dataset are available in the original paper [30].

To assess extinction risk, I used IUCN Red List categories as a standard and widely used proxy [3]. I searched the IUCN Red List database [4] for all 857 species in the chemical defence dataset and recorded the conservation status of all species for which the information was available. This resulted in a final dataset consisting of 809 species from across the amphibian tree of life for which I had data on both extinction risk and chemical defence. I coded extinction risk in two ways. First, as a binary trait (which I term ‘threat’) in which we considered Red List categories least concern (LC) and near threatened (NT) as ‘non-threatened’, and other categories (VU, vulnerable; EN, endangered; CR, critically endangered; EW, extinct in the wild; EX, extinct) as ‘threatened’, in line with recommendations by the IUCN [4]. Second, I coded extinction risk as an ordinal trait (which I term ‘status’) representing increasing levels of threat as follows: 0 = LC, 1 = NT, 2 = VU, 3 = EN, 4 = CR, 5 = EW and EX.

To account for the non-independence of species as data points owing to shared evolutionary history, I took a comparative approach using a recent time-calibrated phylogeny [21]. This was pruned to include only the 809 species for which data were available for both chemical defence and conservation status, and the resulting tree was used for all subsequent analyses. I checked that phylogenetic models fitted the data better than non-phylogenetic equivalents (see electronic supplementary material for details), and finding that they did in all cases I herein report only the comparative methods. The full dataset used for analyses in this study is available at http://dx.doi.org/10.6084/m9.figshare.1399172. All analyses were performed in R v. 3.1.3 [33], using ape [34] for basic manipulation of the phylogeny and other packages as stated for particular methods.

### 2.2. Phylogenetic regression models

I first tested whether threat was predicted by the presence of chemical defence, using phylogenetic logistic regression with the method implemented in phylolm [35]. I then tested whether status was predicted by the presence of chemical defence using phylogenetic generalized estimating equations (GEEs [36]) with a Poisson error structure, also implemented in phylolm [35]. Because treating status as a Poisson trait is perhaps an oversimplification that does not sufficiently reflect its characteristics as an ordinal categorical trait [37], I also analysed this within an ordinal phylogenetic mixed model in a Bayesian MCMC framework, using the MCMCglmm package in R [38]. The analysis was run for 1.1 million generations of which the first 100 000 were conservatively discarded as burn-in and the post-burn-in chain was sampled every 1000 generations.

Other traits have been (or are expected to be) associated with extinction risk in amphibians, and these may confound interpretation of my results if they covary with chemical defence. I therefore used data on latitude and body size of amphibians from reference [35] to check whether these two traits, previously shown to be particularly important predictors of extinction risk [39], are related to chemical defence. As sexual size dimorphism has also been found to predict extinction risk [39], independently of body size, I also calculated an index of sexual size dimorphism as the size of the largest sex divided by that of the smallest to use in another model. There were 288 species for which data were available on latitude, body size and chemical defence. I fitted phylogenetic logistic regression models to test whether these potentially confounding traits were associated with chemical defence (used as the binary response variable in the models). Neither absolute latitude (*p* = 0.611) nor body size (male length, *p* = 0.178; female length, *p* = 0.148) or sexual size dimorphism (*p* = 0.539) predicted the presence of chemical defence in this dataset, and are therefore not considered further for the purposes of this study.

### 2.3. Terminal tip distribution

If chemically defended species have a higher extinction risk, we may also expect that they will be younger than species without such a defence as older lineages are more likely to have gone extinct. I therefore tested this by examining density histograms of the terminal branch lengths of species with and without chemical defences, with branch lengths in bins of 5 million years (Myr). Although this is perhaps a relatively crude approach, because the distribution of terminal branch lengths will also be influenced by factors such as sampling distribution across the tree, we would nevertheless still expect a relative excess of chemically defended species in the youngest age classes if those species are more extinction-prone.

### 2.4. Evolutionary pathway models

The regression-based models in §2.2 test whether chemical defence and extinction risk are linked, and do not assume that the traits evolve along a phylogeny (only that the residuals from the models are phylogenetically structured). This has a benefit as I acknowledge that my proxy of extinction risk, IUCN Red List status, is not an intrinsic biological trait and so does not, in a strict sense, evolve. Nevertheless, such models are limited in their ability to provide inference about cause–effect relationships, which can be evaluated for binary traits using evolutionary pathway models [40]. Therefore, I attempt to use these to test more directly whether the hypothesis that chemical defence leads to an increase in extinction risk is supported, but do so tentatively with this assumption in mind (see electronic supplementary material for justifications of the appropriateness of treating extinction risk as an evolving trait). Such models rely on having multiple independent origins of the traits in question throughout the tree [41], and this is the case for the traits considered herein (electronic supplementary material, figure S1).

I constructed two models and compared their fit, using a likelihood ratio test. The first was a full (eight-parameter) model assuming correlated evolution between threat and chemical defence without imposing constraints on the evolution. The second was a constrained (seven-parameter) model incorporating a single constraint which assumes that chemical defence is gained first which then leads to the lineage becoming threatened, as per reference [40].

### 2.5. Simulations of future fates

In order to provide additional information on the impacts of chemical antipredator defence on the future of amphibian species, I used a simulation approach to extend the results of my previous work on diversification [30]. Specifically, considering a single extant species as a starting point, I simulated 1000 birth–death phylogenies for 5–100 Myr (at 5 Myr intervals). These simulations were performed using TreeSim [42] and represent predictions at regular time points of the fate of a lineage into the future. The birth and death rates were carried out first, using the estimated parameters for non-chemically defended species, and then repeated with the estimates for chemically defended species from reference [30]. At the end of each run, the proportion of the 1000 simulations in which the entire lineage had gone extinct for each time point was recorded.

## 3. Results

**Figure 1.**
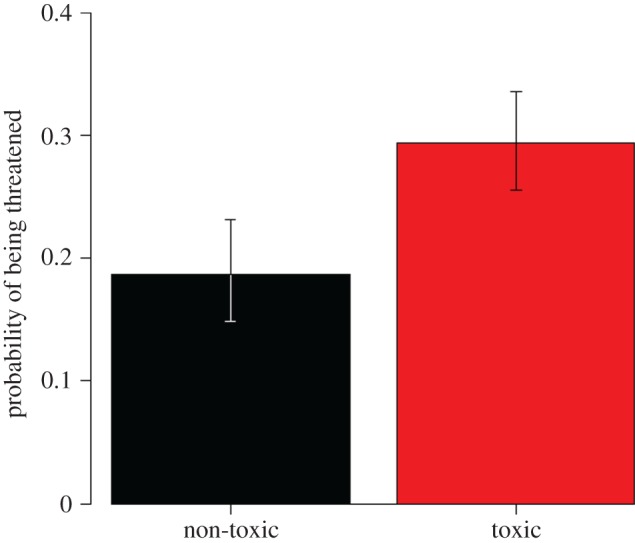
Probability of being classified as threatened for amphibians lacking (black) or possessing (red) chemical defences, based on back-transformed parameter estimates from phylogenetic logistic regression. Error bars are standard errors (also back-transformed from the model). Species with a chemical defence are 60% more likely to be threatened than those without (*p* = 0.002).

**Figure 2.**
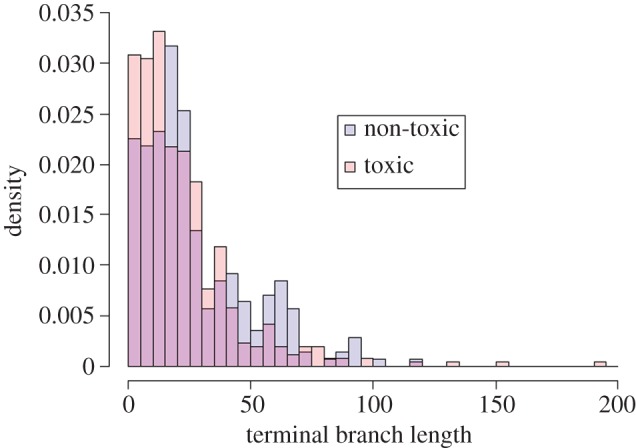
Density histogram of terminal branch lengths for species lacking (blue) or possessing (red) chemical defences. Note the excess of chemically defended species in the youngest three bins (representing terminal branches up to 15 Myr) suggesting that such species are often younger than those lacking a chemical defence.

**Figure 3.**
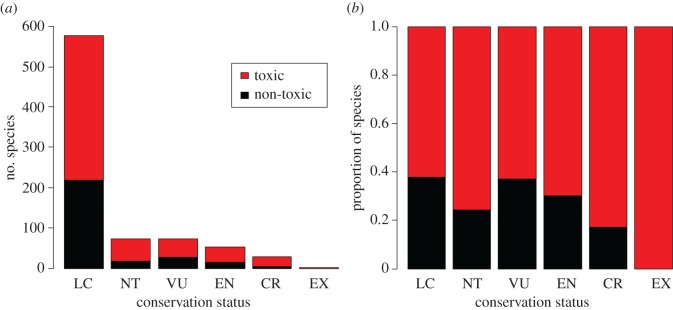
Number (*a*) and proportion (*b*) of species possessing (red) or lacking (black) chemical defence for each IUCN Red List category (severity of extinction risk increases from left to right on the *x*-axis). Although species at higher extinction risk are progressively more likely to possess a chemical defence according to phylogenetic Poisson regression (*p* = 0.024), there is no evidence for this when analysed with an ordinal phylogenetic mixed model (*p* = 0.584).

Contemporary amphibian species which possess chemical defences were 60% more likely to be threatened than species lacking such defences, according to my phylogenetic logistic regression model (*β* = 0.596, s.e. = 0.196, *z* = 3.045, *p* = 0.002; figure 1). Consistent with this, chemically defended species were also overrepresented in the youngest 15 Myr classes of terminal branch lengths (figure 2). My Poisson GEE model of conservation status also found higher extinction risk in chemically defended species when using a more fine-grained measure of threat status (*β* = 0.049, s.e. = 0.022, *z* = 2.263, *p* = 0.024; figure 3); however, this was not the case when status was analysed as an ordinal trait in an MCMCglmm (*β* = 6.707, lower 95% CI = −20.496, upper 95% CI = 33.214, *p* = 0.584).

The evolutionary pathway analyses further corroborate the connection between extinction risk and chemical defence. First, a Pagel's test to test the evolutionary correlation of these two traits found strong support for such a link (likelihood ratio = 16.344, d.f. = 4, *p* = 0.003). Furthermore, when testing the directionality using constrained models, we find strong evidence for a model wherein toxicity is gained first which leads to an increase in extinction risk (likelihood ratio = 11.714, d.f. = 1, *p* = 0.0006).

**Figure 4.**
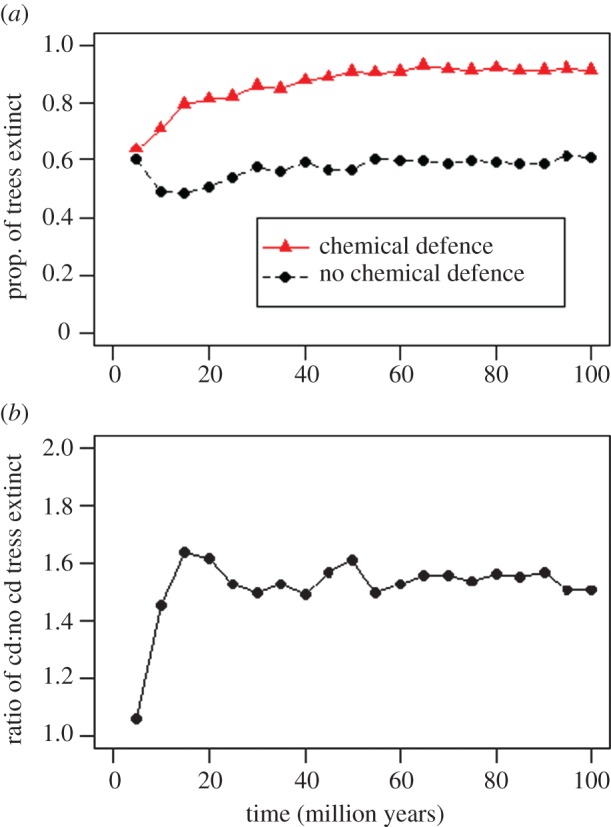
Proportion of 1000 simulated trees that were entirely extinct at each time point (every 5 Myr between 5 and 100 Myr), based on whether diversification parameters used represented species possessing (red triangles) or lacking (black circles) chemical defence (*a*). After 5 Myr, there is little difference in the fate of the lineage, but after between 10 and 15 Myr chemically defended lineages are 50–60% more likely to go extinct, a ratio which then remains stable (*b*).

The results from the simulations suggest that chemically defended species have a higher chance of their entire lineage going extinct than non-chemically defended species (figure 4*a*). Both groups face approximately equal chances of extinction after approximately 5 Myr, but chemically defended amphibians are predicted to have a 50–60% greater chance of complete lineage extinction after 10–15 Myr (figure 4*b*). After this time, the relative risk of the two groups stabilizes (figure 4*b*).

## 4. Discussion

The results generally support the hypothesis that chemical defence is linked to contemporary extinction risk and tentatively support the directional hypothesis that the evolution of chemical defence causes an increased propensity to become threatened. Using a large-scale dataset of amphibians (a taxon of global conservation concern), I demonstrate that chemically defended species are more likely to be threatened and are often younger than non-chemically defended species. Such results are in line with predictions made from a recent study of background extinction rates in amphibians [30] and therefore suggest that such diversification rate estimates can potentially inform estimates of current extinction risk based on species' traits.

It is notable that in the suite of analyses and models used here, all those treating extinction risk as a simple binary categorization (threatened versus not threatened) find support for a relationship with chemical defence. In contrast, the two alternative approaches using the more fine-grained measure of all IUCN Red List categories were less clear. On the one hand, the simplified Poisson model found support for a relationship (though weaker than the binary approaches), whereas the more appropriate [37] ordinal model failed to find any evidence for a link between conservation status and chemical defence. There are two potential explanations for this. First, the lack of relationship from the ordinal mixed model could be a power issue as this was much more parameter-rich than the Poisson model (containing not only a five- to six-category response variable, but also estimated parameters for thresholds between categories). Alternatively, and I suggest more likely given the reasonable sample size of this analysis, the more appropriate way of modelling status [37] revealed that there is indeed no fine-grained relationship between defence and threat status. This does not necessarily conflict with the consistent results from the binary-coded analyses. I interpret this as evidence that chemical defence is related to the probability of being threatened, but the degree of that threat may be controlled by other factors (including the intensity of the drivers of declines such as habitat degradation) that have an overriding influence on more fine-grain levels of severity of extinction risk.

Owing to the large number of species for which IUCN conservation status is unknown, either owing to no assessment being carried out or to insufficient information (i.e. data-deficient species), there is a need for predictive methods if we are to adequately assess the global extinction risk of many groups of organisms. Comparative approaches which investigate traits that are linked to conservation status have been used as a promising approach [7]. Furthermore, a recent paper has suggested that predictive models using such traits are often reasonably reliable and could generate a cost saving of approximately $220 million [43] over assessing all data-deficient species directly. Many aspects of the biology of a particular species are likely to influence the accuracy of these predictions via their effects on extinction risk, and so knowledge of additional traits that can be incorporated into such models should provide an additional benefit.

I wish to stress that I am not advocating chemical defence as the only, or even the most important, trait for predicting extinction risk—many others are also linked to conservation status. For instance, one study [10] found that amphibians experiencing rapid declines more frequently had an aquatic life-stage even when compared with other threatened species, and that species experiencing enigmatic and rapid declines were additionally characterized by low clutch size, environmental variables such as high altitude and stable climates. Another paper [11] found that amphibian extinction risk was associated with low clutch sizes and also larger body size, but concluded that these traits modulate geographical range size, which directly leads to increased extinction risk. However, if we are to predict the extinction risk of species based on traits, then it is essential to use as much information as possible in a comprehensive modelling framework, as inaccuracy can be detrimental to conservation efforts. As a hitherto unstudied trait in this context that predicts a 60% increase in the probability of a species being threatened, I propose that chemical antipredator defence may be a useful addition to the toolbox in some cases. However, it is unlikely to be useful for estimating finer-scale levels of extinction risk, which will limit its use for many practical purposes.

The datasets used for contemporary extinction risk and background extinction rate estimates are independent of each other, but despite this my results using present-day IUCN Red List categories confirm predictions made from a recent macroevolutionary study [30]. This correspondence suggests that studies on evolutionary diversification can give valuable information to shed light on contemporary conservation concerns. I predict that such inferences may be common because species will vary in their susceptibility to extinction as a function of a range of biological attributes. For instance, traits associated with slower life histories are commonly found to be associated with higher extinction risk in a range of taxa facing a variety of threats [10,12,14]. Therefore, I expect that many traits have a consistently detrimental influence on susceptibility to extinction when faced with a variety of specific threats that are driving the population declines, and consequently that associations between traits and extinction may often be temporally stable. This idea is also consistent with the results from my simulations, as they imply that differences in extinction risk between species with and without chemical defences stabilize relatively quickly, suggesting that the response to population decline (whatever the cause) is also relatively stable across time.

The evolutionary pathway models implemented herein indicate that it is the gain of chemical defence that increases the species' susceptibility to extinction, rather than a simple correlation between the two traits. I acknowledge that this conclusion is more tentative than my others as I assume that conservation status is an evolving trait and follows a Markov process, but, nevertheless, I contend that this approach can at least provide suggestive insights. The concordance of the results from models assuming that both traits evolve and those that do not make the assumption (along with previous analyses of background extinction rates [30]) further suggest that similar underlying biological processes may be influencing susceptibility to extinction now as in the past. This is despite the proximate causes of extinction over time, and consequently the scale and rate of contemporary extinction, being very different.

There is a remaining uncertainty as to the mechanistic underpinnings of the influence of chemical defences on the probability of extinction, but the current results shed additional light on the plausibility of the potential mechanisms highlighted in the Introduction. The ‘costly chemical’ and ‘marginal habitats' hypotheses are perhaps poorer explanations for increased extinction risk at both evolutionary and contemporary scales. Reduced competition after population declines should increase the resources available to individuals and therefore ease the energetic trade-offs at the heart of the costly chemical hypothesis. Similarly, the marginal habitats hypothesis relies on the intrinsic vulnerability of low carrying-capacity environments, but a substantial decline in the population should again allow a relatively fast recovery until the carrying capacity is once again reached, owing to reduced competition [44], all else being equal. Nevertheless, the marginal habitats hypothesis would also explain the higher speciation rates of chemically defended amphibians [30] if the move to such habitats is also associated with new ecological opportunities or often simply leads to allopatric speciation. Although different factors may influence speciation versus extinction rates, the marginal habitats hypothesis is the only one that could simultaneously explain both and so may provide a highly plausible explanation for the results here. The ‘slow life-history’ hypothesis predicts that chemical defence should lead to the evolution of slower life histories as a result of reduced extrinsic mortality [45]. Because such traits are also characterized by a slow rate of population growth [17], this is potentially a prime candidate mechanism for a temporally stable relationship between chemical defence and increased extinction risk in the face of many different threats. However, one problem for this hypothesis is that slow life histories may also lead to slower speciation rates in many circumstances owing to longer generation times, but speciation rates were found to be higher in chemically defended amphibians [30].

Overall, I present the first evidence that an antipredator defence is associated with increased contemporary extinction risk in amphibians. I highlight that this conclusion follows a prediction deriving from work on background extinction rates and therefore suggests that patterns of trait-dependent extinction can be conserved across substantial timescales. Finally, I provide tentative evidence that chemical defence is driving the increased susceptibility to extinction in amphibians, rather than the relationship being a product of an incidental correlation, thus shedding further light on our knowledge of the evolutionary consequences of antipredator defence.

## Supplementary Material

Supplementary Material: additional details of the methods used in the paper and Figure S1 (showing multiple estimated transitions in extinction risk throughout the phylogeny).

## Data accessibility

The full dataset used for analyses in this study is available from the Figshare repository: http://dx.doi.org/10.6084/m9.figshare.1399172.
