# Supplementary material for: Chemical antipredator defence is linked to higher extinction risk
Source: R Soc Open Sci. 2016 Nov 23;3(11):160681. doi: 10.1098/rsos.160681 (PMC5180155; doi:10.1098/rsos.160681)
Supplement: Supplementary Material: additional details of the methods used in the paper and Figure S1 (showing multiple estimated transitions in extinction risk throughout the phylogeny). [file rsos160681supp1.docx]

**Supplementary Information**

**Justifications for treating conservation status as an evolving trait**

In one of the analyses in the main paper (the evolutionary pathway models), I treat conservation status as an evolving trait. Despite this not being true in a strict sense - conservation status is a human categorisation rather than in inherent biological trait - I herein offer three justifications to support this assumption as reasonable in this case.

Firstly, a Pagel’s (1994) test for correlated evolution conducted in phytools, which uses pathway modelling, corroborate those from our phylogenetic regression (see Results) by finding evidence for correlated evolution between threat and chemical defence (P=0.003). This is despite the Pagel’s test assuming that both traits evolve and taking into account inferred ancestral transitions, suggesting that it is likely that assuming threat evolves is not strongly misleading.

Secondly, threat exhibits significant phylogenetic signal as measured by Fritz & Purvis’ (2010) D statistic for binary traits (D=0.495, P<0.0002), estimated in caper (Orme *et al*., 2013) based on 5000 permutations. Therefore, extinction risk as measured using IUCN Red List category as a proxy show similar properties to an evolving trait, in that it is more likely to be shared by more closely related species. Taken alongside the first point, this suggests that extinction risk behaves as an evolving trait for the purposes of comparative analyses.

Thirdly, IUCN Red List categories have been widely used as a proxy for extinction risk, which is itself a function of many attributes. Since many of these factors contributing to extinction risk are likely to be evolving traits of the species, extinction risk should be expected to evolve, in a sense, over the phylogeny via the evolution of related variables. Therefore, while Red List categories do not evolve, we consider it reasonable to expect that the underlying extinction risk (which it is intended to represent) should 'evolve' in the sense that it experiences transitions over the phylogeny, as modelled by evolutionary pathway analyses.

**Details of checks to ensure phylogenetic models were more appropriate than non-phylogenetic alternatives**

I checked that phylogenetic logistic regression was justified over standard logistic regression in three ways, all of which provided support for this and so I report only the results from the phylogenetic logistic regressions. Firstly, I fit a standard (non-phylogenetic) logistic regression and compared these alternative models using AIC. I found a ∆AIC value of 61.8 in favour of the phylogenetic model (values greater than ~5 are often considered strong evidence to prefer one model over another). Secondly I examined the α parameter estimated in the phylogenetic model, which ranges between 0 and 1 with low values indicating greater phylogenetic signal. In this model α=0.016, again suggesting that the phylogenetic logistic regression was justified. Finally, I tested whether the residuals from the model exhibited significant phylogenetic signal (as expected if phylogenetic control is necessary) and found this to be true whether using Pagel’s λ (λ=0.653, P=3e-26) or Blomberg’s κ (κ=0.065, P=0.024), both estimated in phytools (Revell, 2012). The Poison-distributed generalised estimating equations used to model 'status' are not likelihood-based and so cannot be compared with other models using information theoretical measures such as AIC, and nor do they fit an α parameter as with the phylogenetic logistic regression models. Nevertheless, I checked that the use of a phylogenetic model was justified here by evaluating the phylogenetic signal in the residuals as above. This was again found to be the case using both Pagel’s λ (λ=0.701, P=2.9e-38) and Blomberg’s κ (κ=0.071, P=0.009).

**References**

Revell, L.J. (2012) phytools: an R package for phylogenetic comparative biology (and other things). Methods in Ecology and Evolution, 3, 217-223.


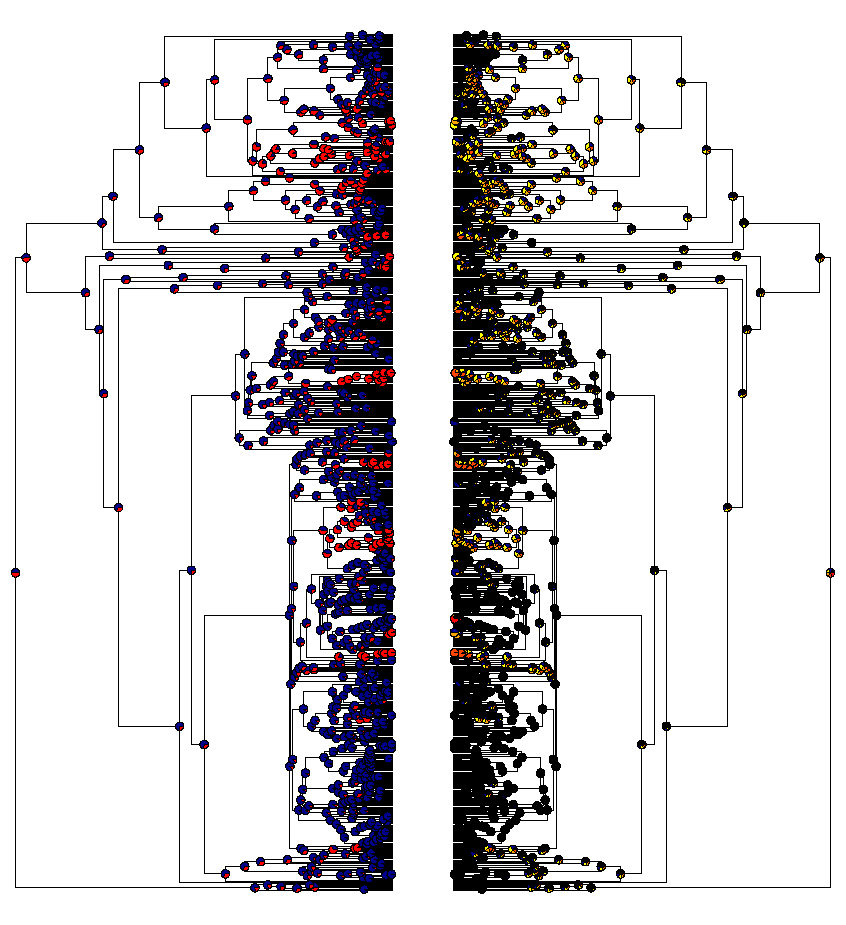


**Figure S1 -** Ancestral state reconstruction of IUCN Red List categories coded as a binary trait (left) and as a multinomial trait with each particular status considered separately (right). In the left hand tree, blue represents non-threatened and red represents threatened. In the right hand tree, warmer colours represent high threat levels (black = Least Concern, blue = Near Threatened, yellow = Vulnerable, light orange = Endangered, dark orange = Critically Endangered, red = Extinct). Ancestral states were estimated under a maximum likelihood framework with an 'all rates different' model (selected as the model with highest log-likelihood) using the 'ace' function in ape^1^. This reconstruction is simply intended to show that threatened categories appear frequently throughout the phylogeny, see ref. 2 for a similar demonstration for chemical defence.

1. Paradis E., Claude J. & Strimmer K. (2004) APE: analyses of phylogenetics and evolution in R language. Bioinformatics, 20, 289-290.

2. Arbuckle, K. & Speed, M.P. (2015) Antipredator defences predict diversification rates. Proceedings of the National Academy of Sciences of the USA, 112, 13597-13602.
